# Detection and Preliminary Genomic Characterization of Poultry-Derived *Salmonella enterica* from Southern Kazakhstan

**DOI:** 10.3390/antibiotics14121195

**Published:** 2025-11-25

**Authors:** Bolat Yespembetov, Zhumagul Kirkimbayeva, Akbope Abdykalyk, Assel Akhmetova, Alexandr Shevtsov, Nazym Syrym, Sabira Alpysbayeva, Makhpal Sarmykova, Azamat Abdimukhtar, Aktoty Anarbekova, Bekzat Yerzhigit, Andrey Shestakov, Nurlan Kozhabergenov, Bekbolat Usserbayev, Yerbol Bulatov, Alinur Toleukhan

**Affiliations:** 1 Research Institute of Biological Safety Problems, 080409 Gvardeyskiy, Kazakhstan; b.yespembetov@biosafety.kz (B.Y.); a.abdykalyk@biosafety.kz (A.A.); n.syrym@biosafety.kz (N.S.); s.alpysbaeva@biosafety.kz (S.A.); m.sarmykova@biosafety.kz (M.S.); a.abdimukhtar@biosafety.kz (A.A.); a.anarbekova@biosafety.kz (A.A.); b.yerzhigit@biosafety.kz (B.Y.); n.kozhabergenov@biosafety.kz (N.K.); b.usserbayev@biosafety.kz (B.U.); ye.bulatov@biosafety.kz (Y.B.); 2 Faculty of Veterinary and Zoo Engineering, Department of Microbiology, Virology and Immunology, Kazakh National Agrarian Research University, 050010 Almaty, Kazakhstan; zhumagul77@yandex.ru; 3 National Center for Biotechnology, 010000 Astana, Kazakhstan; akhmet.assel@gmail.com (A.A.); ncbshevtsov@gmail.com (A.S.); 4 Faculty of Agronomy & Plant Protection, Ulyanovsk State Agrarian University Named After P. A. Stolypin, 432017 Ulyanovsk, Russia; andrewschestakov@yandex.ru

**Keywords:** *Salmonella enterica*, poultry, antimicrobial resistance, disk diffusion, whole-genome sequencing, MLST, QRDR, plasmids, Kazakhstan, One Health

## Abstract

**Background/Objectives**: *Salmonella enterica* is a major cause of foodborne infection globally, with poultry acting as an important reservoir. However, data from Central Asia remain limited. This study provides preliminary phenotypic and genomic characterization of *S. enterica* isolates recovered from poultry farms in southern Kazakhstan, focusing on antimicrobial resistance (AMR), serotypes/sequence types and phylogenetic relationships. **Methods**: In October 2024, 335 poultry and environmental samples were collected from three regions of southern Kazakhstan using a cross-sectional, detection-focused sampling strategy. Isolation of *Salmonella enterica* followed enrichment and selective culturing, with confirmation by biochemical assays, slide agglutination serology and real-time PCR. Antimicrobial susceptibility testing was performed using the Kirby-Bauer disk diffusion method and interpreted according to CLSI veterinary breakpoints (VET01/VET08) and CLSI M100 where veterinary criteria were unavailable. Whole-genome sequencing (Illumina) was used for in silico serotyping, MLST, AMR gene detection, plasmid replicon typing and SNP-based phylogenetic reconstruction. **Results**: Nine *S. enterica* isolates were confirmed (overall yield 2.7%; 9/335), comprising *S.* Enteritidis (ST11; *n* = 4), *S.* Infantis (ST32; *n* = 3) and ST68 *(n* = 2; Choleraesuis/Paratyphi C lineage). All isolates were resistant to ciprofloxacin, and most displayed resistance to ampicillin, gentamicin and trimethoprim-sulfamethoxazole. Plasmid-associated AMR determinants, including *blaTEM-116*, *tet(A)*, *sul1* and *dfrA14*, were frequently identified on IncF-type replicons. Phylogenetic analysis revealed that the isolates clustered with previously described Eurasian poultry-associated lineages. **Conclusions**: In this small, exploratory sample from poultry farms in southern Kazakhstan, all recovered *S. enterica* isolates were multidrug-resistant, with universal fluoroquinolone resistance and frequent plasmid-borne AMR genes. These preliminary findings provide baseline genomic evidence and highlight the need for broader, harmonized AMR surveillance in the regional poultry sector.

## 1. Introduction

*Salmonella enterica* remains one of the leading causes of foodborne illness worldwide, with poultry, meat, and eggs serving as key transmission vehicles. This continues to pose a serious public health and economic challenge in many countries [1,2]. The intensification of poultry production and the increasing complexity of supply chains have created favorable conditions for the emergence and spread of high-risk *Salmonella* lineages along the farm-to-fork continuum [2]. Among the many serovars, *S.* Enteritidis and *S.* Infantis remain consistently linked to poultry reservoirs and are among the most common serovars in human salmonellosis globally [2,3].

The situation is further complicated by antimicrobial resistance (AMR) in *S. enterica*, which limits treatment options and increases the risk of therapeutic failure. Multidrug resistance (MDR) is frequently reported in poultry-associated strains, with resistance to ampicillin, tetracycline, and fluoroquinolones documented across diverse regions, including China and Latin America [4,5,6]. Fluoroquinolone resistance is typically driven by mutations in the quinolone resistance-determining regions (QRDRs) of *gyrA* and *parC*, often in combination with plasmid-mediated mechanisms. β-Lactam resistance ranges from narrow-spectrum *blaTEM* to extended-spectrum β-lactamases (ESBLs) such as *blaCTX-M,* depending on the region. Additional resistance genes such as *tet(A)*, *sul1*, and *dfrA*, as well as class 1 integrons, are commonly carried on IncF-type plasmids that facilitate horizontal gene transfer [1,6,7]. Of particular concern is the spread of the pESI-like megaplasmid in *S.* Infantis, which has been linked to MDR dissemination in poultry sectors across multiple countries [8,9].

While many countries have implemented robust surveillance systems, data from Central Asia remain sparse. In Kazakhstan, limited studies have reported the presence of non-typhoidal *Salmonella* in poultry products, with common resistance to nalidixic acid, ofloxacin, and tetracycline, along with frequent MDR phenotypes [10]. However, high-resolution genomic data on the circulating serovars and sequence types, the genetic context of AMR determinants, plasmid content, and phylogenetic relationships, particularly in southern poultry-producing regions remain limited.

To provide baseline genomic information relevant to this gap, conventional microbiological methods were combined with whole-genome sequencing (WGS) to characterize *Salmonella enterica* isolates from poultry farms in southern Kazakhstan. The aims were to: (i) confirm species identity and determine serovars and multilocus sequence types (MLST); (ii) assess antimicrobial susceptibility using standardized disk diffusion; (iii) identify AMR genes, QRDR mutations, and plasmid replicons; and (iv) contextualize the isolates phylogenetically using international reference genomes. MLST and WGS are widely used to monitor *Salmonella* population structure and trace transmission pathways across hosts and regions [11,12,13].

## 2. Results

### 2.1. Sampling Yield

**Figure 1:**
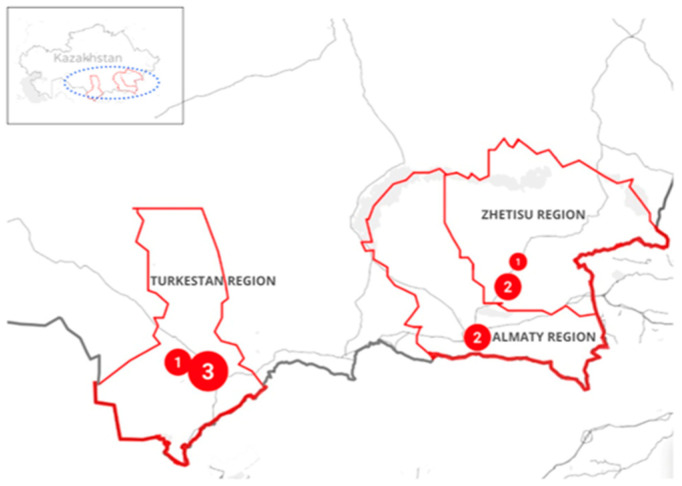
Geographic distribution of sampling regions in southern Kazakhstan. Red circles indicate the regions included in the survey, and the numbers within them show the *Salmonella enterica* isolates recovered from each region.

A total of 335 specimens were collected from poultry and environmental sources across three regions of southern Kazakhstan (Almaty, Zhetisu, Turkestan) (Figure 1). Nine non-duplicate isolates were confirmed as *Salmonella enterica*, corresponding to an overall yield of 2.7% (9/335; 95% CI (Wilson): 1.4–5.0%).

### 2.2. Culture and Preliminary Identification

After pre-enrichment, characteristic black-centered colonies were observed on bismuth sulfite agar, consistent with H_2_S-mediated iron reduction. Subculture on Salmonella-Shigella (SS) agar again yielded black-centered colonies, on XLD agar colonies appeared red-pink with black centers, typical of *Salmonella*. Out of 335 samples, presumptive colonies were detected in 47 (14%) on bismuth sulfite agar, 19 (5.7%) on SS agar, and 9 (2.7%) were confirmed on XLD agar and through follow-up tests.

### 2.3. Microscopy and Biochemical Profile

Gram staining showed Gram-negative rods. On Hiss carbohydrate media, isolates fermented glucose (+), mannose (+), maltose (+) and did not ferment sucrose (−) or lactose (−), consistent with *Salmonella*.

### 2.4. Serology (Slide Agglutination)

Prior to whole-genome sequencing, serological identification was carried out using slide agglutination with commercial polyvalent and monovalent antisera targeting phase 1 and phase 2 antigens. All nine isolates reacted positively with polyvalent O antisera. Based on the agglutination patterns, four isolates exhibited the antigenic formula O:9, H:g,m, consistent with *S.* Enteritidis; three showed O:7, H:r:*1.5*, corresponding to *S.* Infantis; and two displayed O:7, H:c:1.5, characteristic of the *S.* Choleraesuis/Paratyphi C lineage (ST68). These preliminary serotype assignments were subsequently confirmed by in silico analysis using SeqSero2 v1.3.2 (see Section 4.9).

### 2.5. Molecular Identification (Real-Time PCR)

All nine isolates tested positive for *Salmonella enterica* using a commercial real-time PCR assay *(Ct)* < 45, confirming species identity. This molecular confirmation was consistent with the biochemical and serological results described above.

### 2.6. Phenotypic Antimicrobial Susceptibility

Using CLSI interpretive standards (VET01/VET08; M100-2024 where veterinary breakpoints were unavailable) [14,15,16], all isolates were resistant to ciprofloxacin (9/9; 100%). Ampicillin resistance was detected in 7 isolates (77.8%), with 2 (22.2%) showing intermediate susceptibility. No full resistance to chloramphenicol was observed (0/9; 0%), though 2 isolates (22.2%) exhibited intermediate susceptibility. Gentamicin resistance occurred in 8 isolates (88.9%) (one intermediate) and trimethoprim-sulfamethoxazole resistance was also high (8/9; 88.9%).

According to EUCAST ECOFFs for nalidixic acid (wild-type cutoff ≥ 16 mm), 6 isolates (66.7%) were classified as non-wild-type. No statistically significant associations were found between serovar and resistance phenotype (Fisher’s exact test with Benjamini–Hochberg correction; all adjusted *p* > 0.05).

### 2.7. Genotypic AMR Determinants (Conventional PCR)

Conventional PCR revealed a limited distribution of β-lactamase genes: *blaTEM* was detected in 3/9 (33.3%) isolates, while *blaCTX-M*, *blaOXA-1*, and *blaSHV* were not detected. The tetracycline resistance gene *tet(A)* was found in 2/9 (22.2%) while *tet(B)* was absent.

Amplicons of QRDR-associated loci (*gyrA*, *gyrB*, *parC*, *parE*) were successfully obtained in most isolates (9/9, 9/9, 8/9 and 6/9, respectively).

**Figure 2:**
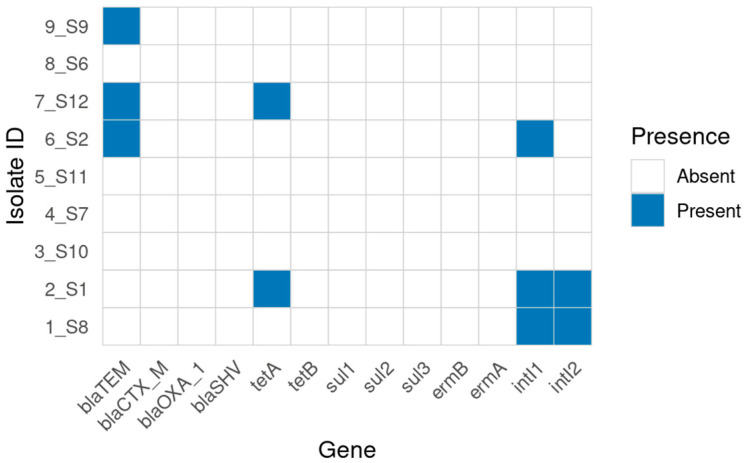
Distribution of antimicrobial resistance (AMR) genes detected by conventional PCR in nine *Salmonella enterica* isolates. Each column represents a resistance gene; each row corresponds to an isolate ID. Blue squares indicate gene presence.

No amplicons were detected for sulfonamide (*sul1*, *sul2*, *sul3)* or macrolide (*ermA*, *ermB*) genes. Class 1 and class 2 integron integrases (*intI1*, *intI2*) were identified in 3/9 (33.3%) and 2/9 (22.2%) isolates, respectively (Figure 2). A summary of the combined phenotype–genotype alignment is provided in Table 1.

### 2.8. Whole-Genome Sequencing and In Silico AMR

**Figure 3:**
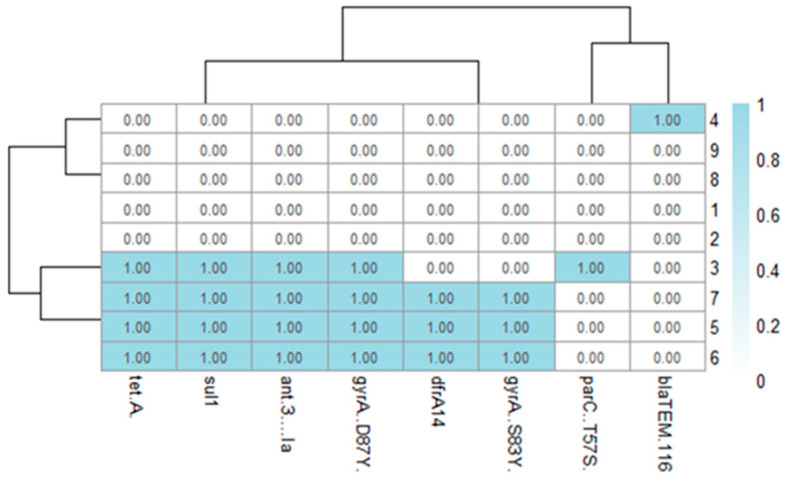
Heatmap of antimicrobial resistance (AMR) genes and point mutations identified by whole-genome sequencing in nine *Salmonella enterica* isolates from poultry in southern Kazakhstan. Rows represent isolate IDs, and columns correspond to specific AMR genes or chromosomal mutations (*tetA*, *sul1*, *ant (3″)-Ia*, *gyrA_D87Y, dfA14*, *gyrA_S83Y*, *parC_T57S*, *blaTEM-116*). Blue shading indicates gene presence (value = 1).

All genome assemblies passed quality control, ranging from 21 to 89 contigs with N50 values between 224,523 and 490,288 bp. Average genome size was around 4.9 Mb with GC content near 52.1%. Whole-genome sequencing confirmed the PCR findings and uncovered additional AMR genes not covered by the PCR panel. The most frequently identified were *tet(A)*, *sul1*, *ant(3″)-Ia*, *dfrA14*, and *blaTEM-116*. Common QRDR mutations included *gyrA_S83Y*, *gyrA_D87Y*, and *parC_T57S*, which aligned with the reduced susceptibility to quinolones observed phenotypically. A summary of gene and point mutations’ presence/absence is shown in Figure 3.

### 2.9. In Silico Serotyping and MLST

Serotyping and MLST identified three distinct *Salmonella* lineages. Four isolates (44.4%) were identified as *S. Enteritidis*, ST11; three (33.3%) as *S. Infantis*, ST32; and two (22.2%) as ST68, within the *S. Paratyphi C/S*. *Choleraesuis/S. Typhisuis* group. Notably, ST68 was found only in environmental samples, such as dust and shoe covers (Table 2).

### 2.10. Phylogenetic Placement

A maximum-likelihood phylogenetic tree, constructed from whole-genome SNPs of the nine study isolates, one reference genome and 113 publicly available genomes representing ST11, ST32 and ST68, revealed three distinct and well-supported clades (Figure S1). ST68 isolates (IDs 8 and 9) clustered with historic *S.* Choleraesuis strains from China (2010). ST32 isolates (IDs 3, 5, 6, and 7) grouped closely with poultry isolates from Russia and Germany (2015–2020). ST11 isolates (IDs 1, 2, and 4) were part of a globally circulating *S.* Enteritidis clade, showing close relatedness to strains from Russia (2020) and South Korea (2011).

### 2.11. Mapping of Antimicrobial Resistance Genes

Annotation of the genome assemblies revealed that most acquired antimicrobial resistance genes were located on plasmid-associated contigs, predominantly linked to IncF-type replicons. Notably, *ant(3″)-Ia*, *sul1*, and *tet(A)* were co-localized on IncFIB(S) plasmids, supporting the hypothesis of plasmid-mediated horizontal transfer of multidrug resistance (MDR) clusters. A summary of AMR genotypes, predicted phenotypes, and plasmid profiles is shown in Supplementary Table S1.

The *blaTEM-116* gene was located on a separate plasmid, while quinolone resistance-determining region (QRDR) mutations in *gyrA* and *parC* were chromosomally encoded. Integron elements (*intI1*, *intI2*) were detected in proximity to several AMR genes, further supporting their role in gene mobilization and dissemination. These findings underscore the dual contribution of mobile genetic elements (plasmids and integrons) and chromosomal point mutations in shaping the antimicrobial resistance landscape of poultry-associated *Salmonella enterica* in southern Kazakhstan.

## 3. Discussion

This study analyzed nine *Salmonella enterica* isolates from poultry farms in southern Kazakhstan, focusing on antimicrobial resistance (AMR), plasmids, sequence types and phylogeny. Although the isolate set was small (*n* = 9) and derived from a limited number of farms, the findings provide useful insight into the regional epidemiology of *Salmonella* and add to the global dataset of poultry-associated strains. In many regions, non-typhoidal *Salmonella* remains one of the most important foodborne zoonotic pathogens, with poultry meat and eggs recognized as major vehicles of transmission to humans [17,18]. Large-scale studies from Brazil, Europe and Asia have shown that poultry-associated *Salmonella* often combine specific serovars, characteristic virulence gene profiles and multidrug resistance, creating a high-risk scenario for both animal health and food safety [19,20,21,22,23].

Within our small collection, *S.* Enteritidis (ST11) and *S.* Infantis (ST32) were the most frequently identified lineages from poultry sources, with two ST68 isolates recovered from environmental samples. This distribution is consistent with previous studies reporting Enteritidis and Infantis as the principal poultry-associated serovars globally and frequent causes of human salmonellosis [3,17,18]. Longitudinal analyses from Brazil demonstrated the long-term persistence of *S.* Infantis in commercial poultry systems over more than 25 years [19], while multiple investigations in poultry-production environments and clinical settings have highlighted *S*. Enteritidis as a major serovar at the poultry-human interface [19,22]. Studies from Türkiye and other regions further showed that *S.* Infantis and *S.* Enteritidis isolated from chickens and turkeys frequently harbor multiple virulence genes that may enhance colonization, invasion and persistence within the host [24,25,26,27]. Although ST68 is relatively uncommon, its detection in environmental samples in this study mirrors sporadic detections reported in several international surveys [17], suggesting a possible ecological niche rather than incidental contamination.

Overall, the presence of *S.* Enteritidis ST11 and *S.* Infantis ST32 in Kazakhstani poultry is in line with the global pattern in which a limited number of successful, epidemiologically important lineages are repeatedly recovered along the poultry production chain.

Genotype-phenotype analysis showed universal fluoroquinolone resistance, associated with QRDR substitutions in *gyrA* (S83Y, D87Y); a *parC* (T57S) substitution was also observed. This resistance profile mirrors mechanisms seen in isolates from China, Russia and Europe [10,28] and similar patterns in northern Kazakhstan suggest that fluoroquinolone-resistant lineages may be more widespread nationally [28]. Clinical and poultry-associated *S.* Enteritidis from China and Thailand frequently display reduced susceptibility or resistance to fluoroquinolones, often linked to combinations of *gyrA* and *parC* mutations [22,29], while data from pediatric diarrheal cases also indicate the circulation of fluoroquinolone-resistant *Salmonella* serovars in human populations [23]. Long-term surveillance in the United States has documented increasing resistance in *S.* Typhimurium recovered along the food chain over two decades [18]. In this context, the universal fluoroquinolone resistance observed in our small collection suggests that similar selection pressures may already be acting in the Kazakhstani poultry sector and that clinically important drugs are at risk of becoming ineffective.

β-lactam resistance determinants were comparatively uncommon: *blaTEM* was detected in 3/9 isolates by PCR and WGS additionally identified *blaTEM-116*; *blaCTX-M* appeared only sporadically. This contrasts with findings from China and Turkey, where ESBLs (including *CTX-M*) are increasingly detected [28,30]. Studies of *S.* Enteritidis clinical isolates from China and Thailand have reported frequent resistance to β-lactams, accompanied by diverse resistance genotypes, including plasmid-mediated β-lactamase genes [22,29]. Similarly, work from wet markets and poultry products has highlighted the co-occurrence of virulence factors and β-lactam resistance in poultry-associated serovars [21,25]. The comparatively low ESBL prevalence in our material may therefore reflect differences in antimicrobial usage patterns, especially with regard to third-generation cephalosporins, but this remains speculative in the absence of formal usage data. Continuous monitoring will be required to determine whether ESBL-producing *Salmonella* lineages emerge or expand in this setting over time.

For sulfonamides and macrolides, *sul2/sul3* and *ermA/ermB* were not detected, whereas *sul1* was identified by WGS in a subset of isolates; these genes are more frequent in larger datasets [31]. Tetracycline resistance was associated with *tet(A),* and aminoglycoside resistance with *ant(3″)-Ia*, in line with common mechanisms reported in poultry-associated *Salmonella* worldwide [18,22,27]. In multiple studies, clinical and food-chain isolates of *S.* Enteritidis, *S.* Typhimurium and *S.* Kentucky have shown concurrent resistance to β-lactams, quinolones, sulfonamides and tetracyclines, often linked to mobile genetic elements [18,25,32]. Although our isolate set is small, the presence of key resistance determinants to several clinically important drug classes indicates that the local *Salmonella* population already carries a substantial AMR burden, even if the overall gene repertoire appears narrower than in some large international collections.

Plasmid replicon typing showed that eight of the nine isolates carried IncF-type replicons, particularly IncFIB(S) and IncFII(S). These plasmids are recognized vectors of multidrug resistance in Enterobacteriaceae [6,33]. AMR loci (*tet(A)*, *sul1*, *ant(3″)-Ia*) co-occurred on contigs bearing IncFIB(S) replicons, consistent with plasmid-associated carriage. Class 1 and class 2 integron integrases (*intI1*, *intI2*) were detected in three and two isolates, respectively, a lower frequency than reported in studies from China and Turkey [19]. Previous work has shown that virulence and resistance genes can be co-located on plasmids or within integron-associated cassettes, facilitating co-selection under antimicrobial pressure [32,34,35,36]. For example, virulence plasmid-encoded genes have been described in *S.* Typhimurium and *S.* Kentucky from chicken carcasses [32], while microarray-based profiling across multiple serovars has documented a broad distribution of virulence loci on mobile elements [36]. The coexistence of IncF-type plasmids, integron integrases and MDR phenotypes in our isolates therefore suggests that the local *Salmonella* population has the genetic infrastructure for further acquisition and reshuffling of AMR and virulence determinants.

Notably, 8/9 isolates (88.9%; MDR defined as resistance to ≥3 antimicrobial classes; 95% CI 56.5–98.0%) were multidrug-resistant (MDR), consistent with high MDR rates (≥70–80%) in larger studies [18,19]. Poultry-derived isolates from Bangladesh, China and other regions have shown similar or higher MDR proportions, often involving serovars *S.* Enteritidis, *S.* Typhimurium, *S.* Kentucky and *S.* Pullorum [22,27,28]. Importantly, several of these studies also demonstrated that virulent and MDR *Salmonella* serovars are recovered not only from animals and carcasses but also from children with diarrhea, underscoring their zoonotic relevance [23,27]. The presence of MDR *S.* Infantis ST32 and *S.* Enteritidis ST11 in our material is therefore concerning, given their known involvement in poultry-associated transmission and outbreaks [2].

Phylogenetic analysis indicated regional genetic relatedness: ST11 and ST32 clustered with recent Russian poultry-derived strains, and ST68 was proximal to Chinese *S.* Choleraesuis references [37]; however, transmission routes or directionality cannot be inferred from genomics alone. Studies of *Salmonella* in free-living birds have shown that wild avifauna can act as reservoirs or vectors of diverse serovars, sometimes carrying strains related to those found in domestic poultry [38]. In addition, work on genotyping methods such as MLVA has illustrated the value of high-resolution subtyping for tracking clonal lineages across animal, food and human sectors [39]. Extending genomic surveillance in Kazakhstan to include wild birds, hatcheries, slaughterhouses, retail meat and human isolates would help to clarify the transmission pathways and the potential role of wildlife or trade networks in disseminating these lineages.

Although we did not characterize virulence genes in our isolates, the combination of MDR phenotypes, IncF plasmids and globally prevalent serovars suggests substantial pathogenic potential. Numerous studies have described the broad distribution of “classic” virulence factors among *Salmonella* spp., including SPI-1- and SPI-2-encoded effectors such as SipA, SopA, SopB, SopD, SopE2 and SifA, as well as plasmid-borne genes such as *spvC* and *pefA* [34,35,36,40]. Poultry-associated *S.* Enteritidis and *S.* Typhimurium often harbor multiple virulence markers, with many strains positive for at least six or more virulence genes [24,25,27]. Recent work on broiler-derived *S.* Enteritidis and *S.* Typhimurium has shown that high positivity for virulence genes provides clear evidence of high pathogenicity and zoonotic risk [41]. By analogy, it is plausible that the MDR *S.* Enteritidis ST11 and *S.* Infantis ST32 lineages identified in this study also carry complex virulence gene repertoires, which warrants further investigation by targeted virulome analysis.

This study has several limitations. First, the sample size was small (*n* = 9) and isolates were recovered from a limited number of farms and time points, so the data cannot be used to estimate prevalence or to capture the full diversity of *Salmonella* circulating in poultry in southern Kazakhstan. Second, the analysis did not include human or retail meat isolates, which would be necessary to directly assess zoonotic transmission along the poultry production chain. Third, we did not assess virulence gene content, biofilm formation or other fitness traits, so our inferences about pathogenic potential rely on comparisons with previously characterized serovars and lineages rather than direct evidence [19,21,26,28,36]. Despite these constraints, the present work provides baseline genomic and phenotypic information on poultry-associated *Salmonella* in this region and situates our findings within the broader international literature on virulence and AMR in *Salmonella*.

Overall, the results reflect global trends: fluoroquinolone resistance, IncF plasmid carriage, and the dominance of ST11/ST32, while also revealing local distinctions, such as low ESBL prevalence and the absence of *sul2/sul3* and *ermA/ermB,* despite the presence of *sul1.* When viewed alongside data from poultry, free-living birds, clinical isolates and food-chain surveillance in other countries [18,19,23,38], our findings support the need for integrated One Health surveillance that links farm-level interventions, antimicrobial stewardship and public health monitoring in Kazakhstan.

## 4. Materials and Methods

### 4.1. Sampling and Ethics

Ethical approval for sample collection was obtained from the Bioethics Committee of the Research Institute for Biological Safety Problems, Kazakhstan (Protocol No. 4; 15 November 2023). Non-invasive sampling and routine post mortem collection were conducted with owner consent; no experimental procedures were performed on live animals.

### 4.2. Case Selection and Sampling

A cross-sectional convenience sampling strategy was used to detect and characterize *Salmonella enterica* isolates rather than to estimate prevalence. Field work was conducted in October 2024 across three southern regions (Almaty, Zhetisu, Turkestan). Within each region, three poultry-producing rural areas were selected based on site access, willingness to participate and cold-chain feasibility. In total, 335 specimens were collected, including cloacal swabs (168), tracheal swabs (68), fresh feces (20), organ tissues from routine mortalities (25) and environmental swabs (dust, surfaces, shoe covers) (54). Specimens were transported at 2–8 °C and processed within 24 h. Region-level counts and specimen-type distribution are summarized in Supplementary Table S2.

### 4.3. Bacterial Isolation

Isolation of Salmonella followed procedures described in ISO 657-1:2017; FDA BAM Chapter 5 [42,43,44]. Samples were inoculated into GRM nutrient broth (FBRI SRCAMB, Obolensk, Russia) for pre-enrichment and incubated at 37 °C for 24 h. Enriched cultures were streaked onto Salmonella-Shigella agar (Condalab, Madrid, Spain), bismuth sulfite agar (Condalab, Madrid, Spain) and xylose lysine deoxycholate (XLD) agar (Condalab, Madrid, Spain) and incubated at 37 °C for 24 h. Presumptive *Salmonella* formed black-centered colonies consistent with H_2_S-mediated iron reduction [42,43].

### 4.4. Microscopic Examination

Gram staining was performed using a commercial Gram staining kit (Condalab, Madrid, Spain) according to the manufacturer’s instructions.

### 4.5. Molecular Identification (Real-Time PCR)

Real-time PCR confirmation of *Salmonella enterica* was performed using the VetMAX™ *Salmonella enterica* Detection Kit (Applied Biosystems™, Thermo Fisher Scientific, Waltham, MA, USA), according to the manufacturer’s instructions. Each run included positive extraction controls, no-template controls, and internal amplification controls to verify assay performance. A sample was considered positive when a sigmoidal amplification curve appeared in the FAM channel with a cycle threshold (Ct) < 45 and a valid internal control signal.

### 4.6. Slide Agglutination Serology

Slide agglutination was performed using commercial O and H antisera (phases 1 and 2; AO “EKOlab,” Elektrougli, Moscow Region, Russia). Reactions were observed at 1–2 min on a white tile under adequate lighting. Antigenic profiles were interpreted following the White–Kauffmann–Le Minor scheme [14]. Negative controls (no antisera) were included in each reaction to exclude nonspecific agglutination.

### 4.7. Antimicrobial Susceptibility Testing

Antimicrobial susceptibility was determined by the Kirby–Bauer disk diffusion method on Mueller–Hinton agar and interpreted using CLSI veterinary breakpoints (VET01/VET08) and CLSI M100 (2024) where veterinary criteria were unavailable [15,16,45]. The antibiotic panel reflected agents relevant to poultry production and CLSI interpretive categories: ampicillin 10 µg, chloramphenicol 30 µg, cefotaxime 30 µg, trimethoprim-sulfamethoxazole 25 µg, ciprofloxacin 5 µg, gentamicin 10 µg, nalidixic acid 30 µg. Suspensions were adjusted to 0.5 McFarland (1–2 × 10^8^ CFU/mL), lawn-inoculated then incubated at 35 ± 2 °C for 16–18 h. Zone diameters were measured in millimeters; a 6 mm zone was recorded for confluent growth. Quality control was performed using *Escherichia coli* ATCC 25922. Where applicable (for nalidixic acid), EUCAST epidemiological cut-off values (ECOFFs) were applied to distinguish wild-type from non-wild-type phenotypes [46].

### 4.8. AMR Gene Screening (Conventional PCR)

Conventional PCR was used to detect genes conferring resistance to β-lactams (*blaTEM*, *blaSHV*, *blaOXA-1*, *blaCTX-M*), tetracyclines (*tetA*, *tetB*), sulfonamides (*sul1*, *sul2*, *sul3*), macrolides (*ermA*, *ermB*), and class 1 and class 2 integrons (*intI1*, *intI2*) (Table 3).

Each 25 µL reaction contained 1× Standard Taq Buffer (New England Biolabs (NEB), Ipswich, MA, USA), 200 µM dNTPs, 0.5 µM of each primer, 1 U Taq DNA polymerase, and 2 µL of template DNA (50–100 ng). Cycling conditions were: 95 °C for 5 min, followed by 30 cycles of 94 °C for 30 s, 55–60 °C for 30 s, and 72 °C for 60 s, with a final extension at 72 °C for 7 min. Amplicons were separated on 1.5% agarose gels stained with ethidium bromide and visualized under UV illumination. All reactions were single-plex.

### 4.9. Whole-Genome Sequencing and De Novo Assembly

Genomic DNA was extracted using the innuPREP DNA Mini Kit 2.0 (Analytik, Jena, Germany) according to the manufacturer’s protocol. Libraries were prepared with the Collibri ES DNA Library Prep Kit (Invitrogen, Carlsbad, CA, USA, Cat. A38607096) and sequenced on an Illumina MiSeq using the MiSeq Reagent Kit v3 (600-cycle) (San Diego, CA, USA, MS-102-3003) to generate 2 × 300 bp reads.

Reads were trimmed with Trimmomatic v0.39 [56] using LEADING:3, TRAILING:3, SLIDINGWINDOW:4:25, MINLEN:36, and quality evaluated using FastQC (v. 0.11.9) [57]. Draft assemblies were generated using SPAdes v3.15.5 [58] with—careful mode and evaluated with QUAST [59]. Assemblies were retained only if 4.0–6.5 Mb, N50 ≥10 kb, and <1000 contigs. Assembly quality metrics for all *Salmonella enterica* isolates were assessed using Quast (Version 5.3.0) and checkm2 (Version 1.1.0) tools (Supplementary Table S3). Because short-reads can fragment plasmids, AMR calls were cross-validated with AMRFinderPlus v3.12-2024-05-02.2 and staramr (v. 0.9.1) to minimize false negatives.

### 4.10. In Silico Serotyping and MLST

Serovars were predicted using SeqSero2 [28]. MLST was performed using the MLST tool [60] against EnteroBase/PubMLST schemes.

### 4.11. Genotypic AMR Prediction

AMR determinants were predicted using staramr (Public Health Agency of Canada, National Microbiology Laboratory, Winnipeg, MB, Canada, PHAC-NML) [61], integrating ResFinder, PointFinder and PlasmidFinder (≥98% identity and ≥52% length for ResFinder; ≥95% for PointFinder). To reduce false-negative detection, AMR calls were cross-validated using AMRFinderPlus v3.12-2024-05-02.2 [60] in nucleotide mode with Salmonella_enterica organism parameters.

### 4.12. Selection of Publicly Available Reference Genomes

A total of 113 complete *Salmonella enterica* genomes (Supplementary Table S4) were obtained from the publicly available dataset compiled by Cherchame et al. (2022) [62], which was created using the SalmoDEST tool for collecting and filtering complete reference genomes from GenBank. This collection includes only genomes that meet a minimum 50× sequencing coverage and a validated serotype measurement. The present analysis includes genomes corresponding to Enteritidis, Infantis and Paratyphi C detected among our isolates and used as references for phylogenetic comparison and AMR gene localization.

### 4.13. SNP Calling and Recombination Masking

Nine study isolates, *Salmonella* Typhimurium LT2 [63] and 113 publicly available genomes [64] were compared. SNPs were identified using Snippy v4.6.0 [60]. Core genome SNP alignments were generated using snippy-core and recombination masked with Gubbins v3.2.1 [65].

### 4.14. Phylogenetic Analysis

Maximum-likelihood phylogenies were reconstructed using IQ-TREE v2.4.0 [66] with ModelFinder and 1000 ultrafast bootstrap replicates. Visualization and annotation were performed using iTOL v7.2.1 [67].

### 4.15. AMR Gene Presence/Absence Visualization

AMR gene presence/absence across all genomes (*n* = 123) was profiled with AMRFinderPlus and visualized in R v3.6.0 [68] using tidyverse (v. 1.3.2) [69], ape (v. 5.6.2) [70], and ggtree (v. 3.8.2) [71].

### 4.16. Statistical Analysis

Categorical variables were summarized as counts and percentages and continuous variables as medians with interquartile ranges (IQRs). Proportions are reported with 95% confidence intervals (Wilson’s method). Group comparisons used Fisher’s exact tests with Benjamini–Hochberg correction for multiple testing; adjusted *p* < 0.05 was considered statistically significant. All analyses were performed in R v3.6.0.

### 4.17. Use of Generative AI

Use of Generative AI: Where applicable, we disclose that a generative AI assistant (OpenAI ChatGPT v 5.1, November 2025 release) was used only to improve the clarity, consistency and formatting of the manuscript text and to help standardize method descriptions. No data, analyses, figures, or results were generated by AI. All scientific content, data interpretation, statistical analyses, and final text were critically reviewed and approved by the authors prior to submission.

## 5. Conclusions

This study provides genomic and phenotypic insight into a small set of *Salmonella enterica* isolates from poultry farms in southern Kazakhstan. The predominance of poultry-associated serovars such as *S.* Enteritidis ST11 and *S.* Infantis ST32, together with a very high proportion of multidrug-resistant isolates and universal fluoroquinolone resistance, indicates that local poultry flocks harbor lineages with recognized zoonotic potential. The coexistence of MDR phenotypes with IncF-type plasmids and integron integrases suggests that these strains have the genetic capacity for further acquisition and dissemination of resistance determinants, even though extended-spectrum β-lactamase genes were detected only sporadically. Taken together, these findings highlight both convergence with global trends and a potential window of opportunity for intervention: strengthening antimicrobial stewardship in the poultry sector, expanding routine whole-genome sequencing of animal, food and human isolates, and incorporating future virulence gene profiling will be essential to prevent the emergence and spread of highly resistant, highly virulent *Salmonella* clones in Kazakhstan.

## Figures and Tables

**Table 1:** Phenotype–genotype (PCR) comparison for *Salmonella enterica* isolates (disk diffusion vs. conventional PCR).

ID	AMP/β-Lactamase (*blaTEM*, *blaCTX-M*, *blaOXA-1*, *blaSHV*)	CHL	GEN	SXT/Sulfonamide (*sul1*, *sul2*, *sul3*)	TET (*tetA/tetB*)	Integrons (*intI1*, *intI2*)
1_S8	R/-	S/-	R/-	R/-	-	*intI1*^+^, *intI2*^+^
2_S1	R/-	I/-	R/-	R/-	*tet A* ^+^	*intI1* ^+^ *, intI2* ^+^
3_S10	I/-	S/-	R/-	R/-	-	-
4_S7	I/-	S/-	R/-	R/-	-	-
5_S11	R/-	I/-	R/-	R/-	-	-
6_S2	R/*blaTEM*^+^	S/-	R/-	R/-	-	-
7_S12	R/*blaTEM*^+^	S/-	I/-	R/-	*tet A* ^+^	-
8_S6	R/-	S/-	R/-	R/-	-	*intI1*^+^, *intI2*^+^
9_S9	R/*blaTEM*^+^	S/-	R/-	S/-	-	-

AMP—ampicillin; CHL—chloramphenicol; GEN—gentamicin; SXT—trimethoprim—sulfamethoxazole; TET—tetracycline; R—resistant; I—intermediate; S—susceptible (interpreted according to CLSI VET01/VET08 and M100, 2024); “+” indicates gene detected, “-” not detected.

**Table 2:** In silico serotyping (SeqSero2) and MLST typing results for nine *Salmonella* isolates.

Sample ID	Predicted Antigenic Profile	Predicted Serotype	MLST Type	Sample Type
Salm-Otar-1_S8	9:g,m	Enteritidis	11	Localized flushing
Salm-Otar-2_S1	9:g,m	Enteritidis	11	Chicken droppings
Salm-Otar-3_S10	7:r:1.5	Infantis	32	Chicken droppings
Salm-Otar-4_S7	9:g,m	Enteritidis	11	Localized flushing
Salm-Otar-5_S11	7:r:1.5	Infantis	32	Fallen bird
Salm-Otar-6_S2	7:r:1.5	Infantis	32	Fallen bird
Salm-Otar-7_S12	7:r:1.5	Infantis	32	Cloacal swabs
Salm-Otar-8_S6	7:c:1.5	Paratyphi C or Choleraesuis or Typhisuis	68	Dust sample from a private poultry farm
Salm-Otar-9_S9	7:c:1.5	Paratyphi C or Choleraesuis or Typhisuis	68	Sample from shoe covers from a private poultry farm

**Table 3:** Primers used for PCR detection of antimicrobial resistance genes.

Antibiotic Class	Gene	Primer Sequence (5′-3′)	Size (bp)	Ref.
β-lactams	*blaTEM*	F: ATCAGTTGGGTGCACGAGTG R: ACGCTCACCGGCTCCAGA	608	[47]
	*blaOXA-1*	F: ATGAAAAACACAATACATATCAAC R: AAAGGACATTCACGCCTGTG	768	[47]
	*blaCTX-M*	F: TTTGCGATGTGCAGTACCAGTAA R: CCGCTGCCGGTCTTATC	550	[48]
	*blaSHV*	F: TCGCCTGTGTATTATCTCCC R: CGCAGATAAATCACCACAATG	768	[48]
Tetracyc- lines	*tetA*	F: GGTTCACTCGAACGACGTCA R: CTGTCCGACAAGTTGCATGA	577	[49]
	*tetB*	F: CATTAATAGGCGCATCGCTG R: TGAAGGTCATCGATAGCAGG	930	[50]
Sulfonamides	*sul1*	F: CTTCGATGAGAGCCGGCGGC R: GCAAGGCGGAAACCCCGCC	432	[51]
	*sul2*	F: GCGCTCAAGGCAGATGGCATT R: GCGTTTGATACCGGCACCCGT	293	[51]
	*sul3*	F: CATTCTAGAAAACAGTCGTAGTTCG R: CATCTGCAGCTAACCTAGGGCTTTGGA	789	[52]
Macrolides	*ermB*	F: GAAAAGGTACTCAACCAAATA R: AGTAACGGTACTTAAA TTGTTTAC	636	[53]
	*ermA*	F: CTTCGATAGTTTATTAATATTAGT R: TCTAAAAAGCATGT AAAAGAA	645	[53]
Integrons	*IntI1*	F: CAGTGGACATAAGCCTGTTC R: CCCGAGGCATAGACTGTA	160	[54]
	*IntI2*	F: TTATTGCTGGGATTAGGC R: ACGGCTACCCTCTGTTATC	233	[55]

## Data Availability

Raw reads and draft assemblies have been deposited under BioProjects PRJNA1344273, PRJNA1338795 and PRJNA1338608. Records are currently under embargo and will be made public upon acceptance. Per-isolate sample, read and assembly accessions (SAMN/SRR/GCA) are available in the corresponding NCBI BioProject records.

## Supplementary Materials

The following supporting information can be downloaded at https://www.mdpi.com/article/10.3390/antibiotics14121195/s1. Figure S1: Phylogenetic tree of *Salmonella enterica* isolates sequenced in this study compared with global references. ML phylogeny from whole-genome SNPs for 9 study isolates, the LT2 reference, and 113 public genomes (ST11/32/68). Kazakhstan isolates (colored) form three clades; IDs 8–9 cluster with a *S.* Choleraesuis genome from China (2010). Table S1. Summary of the combined phenotype–genotype alignment for *S.* enterica isolates. This corresponds to Table 1 in the main text. Table S2. Distribution of poultry and environmental specimens collected in October 2024. The table lists the number of cloacal swabs, tracheal swabs, fresh feces, organ tissues, and environmental swabs (dust, surfaces, shoe covers) collected in different regions and villages. Table S3. Detailed genome assembly quality assessment results of nine *S.* enterica isolates using Quast and CheckM2. Metrics include number of contigs at various lengths, total length, GC content, N50, N90, auN, L50, L90, number of N’s per 100 kbp, completeness, and contamination. Table S4. *S.* enterica isolates retrieved from public databases (according to Cherchame et al., 2022 [62]). The table includes accession numbers, serotypes, strains, locations, and year of isolation.

## Abbreviations

The following abbreviations are used in this manuscript:

AMR	antimicrobial resistance
AST	antimicrobial susceptibility testing
bp	base pairs
CFU	colony-forming units
CLSI	Clinical and Laboratory Standards Institute
Ct	cycle threshold
ECOFF	epidemiological cut-off value
EnteroBase	public bacterial population genomics platform
GC	guanine–cytosine content
Gubbins	recombination-aware phylogeny software
iTOL	Interactive Tree Of Life (phylogeny visualization)
IQ-TREE	maximum-likelihood phylogenetic inference software
MHA	Mueller–Hinton agar
MLST	multilocus sequence typing
N50	assembly N50 (contiguity metric)
PCR	polymerase chain reaction
PlasmidFinder	database/tool for plasmid replicons
PointFinder	database/tool for chromosomal AMR mutations
PubMLST	public MLST database
qPCR	real-time polymerase chain reaction
QRDR	quinolone resistance–determining region
QUAST	genome assembly quality assessment tool
ResFinder	database/tool for acquired AMR genes
SeqSero2	WGS-based *Salmonella* serotyping software
SNP	single-nucleotide polymorphism
Snippy	bacterial variant calling pipeline
SPAdes	de novo genome assembler
ST	sequence type
WGS	whole-genome sequencing
ZOI	zone of inhibition
