# Supplementary material for: Detection and Preliminary Genomic Characterization of Poultry-Derived Salmonella enterica from Southern Kazakhstan
Source: Antibiotics (Basel). 2025 Nov 25;14(12):1195. doi: 10.3390/antibiotics14121195 (PMC12729890; doi:10.3390/antibiotics14121195)
Supplement: Supplementary file 1 [file antibiotics-14-01195-s001.zip › Supplementary tables.pdf]

Supplementary Table S2. Distribution of poultry and environmental specimens collected in October 2024.

No.	Region	Rural area / Village	Cloacal swabs	Tracheal swabs	Fresh feces	Organ tissues	Environmental swabs (dust, surfaces, shoe covers)	Subtotal
1	Almaty	Belbulak village, Talgar district	12	5	2	2	4	25
2		Kyzylkairat village, Talgar district	23	5	2	4	6	40
3		Kegen rural district, Kegen district	10	5	1	1	3	20
4	Zhetisu	Saryozek village, Konaev agglomeration (Koksu district)	12	6	2	2	-	22
5		Balpyk Bi village, Koksu district	18	7	2	3	-	30
6		Saryozek rural district, Kerbulak district	12	5	-	1	-	18
7	Turkestan	Shauilder village, Otyrar district	28	10	4	4	8	54
8		Temirlan village, Ordabasy district	30	15	4	4	8	61
9		Orangai rural district, Sauran district	23	10	3	4	8	48
		Total	168	68	20	25	54	335

Supplementary Table S3. Detailed genome assembly quality assessment results (using Quast and checkm2).

**Assembly**	**Salm-Otar-1_S8**	**Salm-Otar-2_S1**	**Salm-Otar-3_S10**	**Salm-Otar-4_S7**	**Salm-Otar-5_S11**	**Salm-Otar-6_S2**	**Salm-Otar-7_S12**	**Salm-Otar-8_S6**	**Salm-Otar-9_S9**
# contigs (>= 0 bp)	51	47	103	154	94	116	118	86	66
# contigs (>= 1000 bp)	23	19	37	42	35	42	37	48	43
# contigs (>= 5000 bp)	16	15	28	19	28	32	29	33	32
# contigs (>= 10000 bp)	15	14	26	14	27	31	28	30	30
# contigs (>= 25000 bp)	15	14	22	14	22	26	24	28	28
# contigs (>= 50000 bp)	13	12	20	12	19	20	21	21	21
Total length (>= 0 bp)	4712914	4683210	4904053	4816564	4936562	4940327	4942489	4767194	4757500
Total length (>= 1000 bp)	4702969	4674039	4885999	4767568	4917686	4914697	4915673	4749102	4747616
Total length (>= 5000 bp)	4686849	4666378	4867953	4720178	4902567	4894604	4898806	4711225	4719714
Total length (>= 10000 bp)	4680720	4660249	4853588	4682553	4896927	4888964	4893166	4692107	4708361
Total length (>= 25000 bp)	4680720	4660249	4776651	4682553	4797012	4791560	4817463	4651384	4675991
Total length (>= 50000 bp)	4595967	4592640	4712767	4618102	4685640	4575793	4722097	4377214	4401824
# contigs	29	21	43	89	44	57	48	64	50
Largest contig	1549500	1549499	1179886	1548814	1195360	1195360	1195360	529847	529847
Total length	4706351	4675164	4890233	4798100	4924156	4925011	4923246	4759247	4751934
GC (%)	52.13	52.15	52.17	52.01	52.15	52.15	52.15	52.14	52.14
N50	491923	490295	444544	490288	437110	437405	437110	224523	224523
N90	153358	153190	76084	153155	76392	57642	67889	82582	82582
auN	760552.4	752155.9	496533.0	764128.4	495379.3	480897.6	481600.2	248039.7	250265.8
L50	3	3	4	3	4	4	4	8	8
L90	9	9	16	9	15	18	17	20	20
# N's per 100 kbp	0.00	0.00	0.00	0.00	0.00	0.00	0.00	0.00	0.00
Completeness (checkm2)	100.0	100.0	100.0	100.0	100.0	100.0	100.0	100.0	100.0
Contamination (checkm2)	0.1	0.09	0.14	0.11	0.05	0.08	0.75	0.05	0.05

Supplementary Table S4. *S. enterica* isolates retrieved from database (according to Cherchame et al., 2022).

#	Accession number	Serotype	Strain	Location	Year
1	GCF_000009505.1	Enteritidis	strain	EBI	2010
2	GCF_000280315.2	Enteritidis	strain	USA	1977
3	GCF_000329365.2	Enteritidis	strain	USA	2010
4	GCF_000335875.2	Enteritidis	strain	Mexico	2015
5	GCF_000612325.1	Enteritidis	strain	USA	2013
6	GCF_000623055.1	Enteritidis	strain	Canada	2014
7	GCF_000623095.1	Enteritidis	strain	Canada	2016
8	GCF_000623115.2	Enteritidis	strain	Canada	
9	GCF_000623135.1	Enteritidis	strain	Canada	
10	GCF_000623195.2	Enteritidis	strain	Canada	2011
11	GCF_000623295.1	Enteritidis	strain	Canada	2004
12	GCF_000623315.1	Enteritidis	strain	Canada	2004
13	GCF_000623335.1	Enteritidis	strain	Canada	2009
14	GCF_000623355.1	Enteritidis	str	Canada	2003
15	GCF_000623395.2	Enteritidis	strain	USA	1998
16	GCF_000623455.2	Enteritidis	strain	Canada	1998
17	GCF_000623475.1	Enteritidis	strain	Canada	1999
18	GCF_000623615.2	Enteritidis	strain	Canada	1996
19	GCF_000623655.2	Enteritidis	strain	Canada	1997
20	GCF_000623675.2	Enteritidis	strain	Canada	1997
21	GCF_000623715.2	Enteritidis	strain	Canada	1998
22	GCF_000623735.2	Enteritidis	strain	Canada	1998
23	GCF_000624055.2	Enteritidis	strain	Canada	2009
24	GCF_000624175.1	Enteritidis	strain	Canada	2016
25	GCF_000624395.2	Enteritidis	strain	Canada	2010
26	GCF_000625855.1	Enteritidis	strain	Canada	2008
27	GCF_000626095.2	Enteritidis	strain	Canada	2010
28	GCF_000626175.1	Enteritidis	strain	Canada	2010

29	GCF_000626195.1	Enteritidis	strain	Canada	2006
30	GCF_000626235.1	Enteritidis	str	Canada	2005
31	GCF_000626275.2	Enteritidis	strain	Canada	2008
32	GCF_000626315.1	Enteritidis	str	Canada	2010
33	GCF_000626335.1	Enteritidis	strain	Canada	2011
34	GCF_000626375.1	Enteritidis	strain	Canada	2011
35	GCF_000626695.2	Enteritidis	strain	Canada	2010
36	GCF_000742815.1	Choleraesuis	strain	China	2010
37	GCF_000750215.1	Enteritidis	strain	Canada	2010
38	GCF_000750255.1	Enteritidis	strain	Canada	2010
39	GCF_000750295.1	Enteritidis	strain	Canada	2009
40	GCF_000750335.1	Enteritidis	strain	Canada	2010
41	GCF_000750375.1	Enteritidis	strain	Canada	2010
42	GCF_000750395.2	Enteritidis	strain	Canada	2000
43	GCF_000750415.2	Enteritidis	strain	Canada	2000
44	GCF_000750435.1	Enteritidis	strain	Canada	2009
45	GCF_000750455.1	Enteritidis	strain	Canada	2008
46	GCF_000750475.1	Enteritidis	strain	Canada	2010
47	GCF_000750495.1	Enteritidis	strain	Canada	2010
48	GCF_000783815.2	FDAARGOS		USA	2018
49	GCF_000953495.1	Infantis		EBI	2014
50	GCF_001305235.1	Enteritidis	strain	USA	
51	GCF_001484025.1	Enteritidis	strain	Denmark	2016
52	GCF_001647045.1	Enteritidis	strain	Canada	2000
53	GCF_001705055.1	FORC		USA	2019
54	GCF_001931555.1	Infantis	strain	USA	2015
55	GCF_001931575.1	Infantis	strain	USA	2015
56	GCF_001931595.1	Infantis	strain	USA	2014
57	GCF_001931615.1	Infantis	strain	USA	2014
58	GCF_001973275.2	Enteritidis	strain	USA	2017

59	GCF_002128365.1	Enteritidis	strain	USA	2017
60	GCF_002128385.1	Enteritidis	strain	USA	2013
61	GCF_002220345.1	Enteritidis	strain	Korea	2014
62	GCF_002313085.1	FORC	Korea	Korea	2016
63	GCF_002313105.1	Enteritidis	strain	Korea	2015
64	GCF_002760915.1	Enteritidis	strain	FDA	1956
65	GCF_002760935.1	Enteritidis	strain	USA	1970
66	GCF_002760955.1	Enteritidis	strain	USA	1974
67	GCF_002760975.1	Enteritidis	strain	USA	1977
68	GCF_002760995.1	Enteritidis	strain	USA	1979
69	GCF_002761015.1	Enteritidis	strain	USA	1981
70	GCF_002761035.1	Enteritidis	strain	USA	1981
71	GCF_002761075.1	Enteritidis	strain	USA	1993
72	GCF_002761095.1	Enteritidis	strain	USA	1995
73	GCF_002761135.1	Enteritidis	strain	USA	1969
74	GCF_002761155.1	Enteritidis	strain	USA	1981
75	GCF_002763415.1	Enteritidis	strain	USA	1981
76	GCF_002813935.1	Typhimurium	strain	Korea	2016
77	GCF_002813975.1	Enteritidis	strain	China	2010
78	GCF_002813995.1	Enteritidis	strain	China	2010
79	GCF_002946675.1	MFDS1004839	Korea	Korea	2014
80	GCF_002982095.1	Enteritidis	strain	Brazil	2001
81	GCF_002999175.1			Germany	2015
82	GCF_003031995.1	Enteritidis	strain	Switzerland	
83	GCF_003032035.1	Enteritidis	strain	Switzerland	2018
84	GCF_003184325.1	Enteritidis	strain	Canada	2001
85	GCF_003184425.1	Enteritidis	strain	USA	2001
86	GCF_003312565.1	Enteritidis	strain	USA	2018
87	GCF_003429365.1	Enteritidis	strain	Korea	2017
88	GCF_003515965.1	FORC			

89	GCF_003691385.1	Enteritidis	strain	China	2016
90	GCF_003710165.1	Enteritidis	strain	Canada	2001
91	GCF_004135835.1	FORC		Korea	2002
92	GCF_005889955.1	Enteritidis	strain	USA	
93	GCF_005889975.1	Enteritidis	strain	USA	2019
94	GCF_006517015.1	Enteritidis	strain	China	2013
95	GCF_006517055.1	Enteritidis	strain	Canada	2014
96	GCF_008313815.1	Enteritidis	strain	Denmark	2019
97	GCF_009884355.1	Enteritidis	strain	Korea	2011
98	GCF_010919335.1	Infantis	strain	Israel	2008
99	GCF_012049805.1	Enteritidis	strain	China	2013
100	GCF_012050205.1	Enteritidis	strain	China	2013
101	GCF_012050385.1	Enteritidis	strain	China	2013
102	GCF_012050525.1	Enteritidis	strain	USA	2013
103	GCF_012050745.1	Enteritidis	strain	China	2012
104	GCF_012050905.1	Enteritidis	strain	China	2012
105	GCF_013377315.1	Infantis	strain	Vietnam	2017
106	GCF_013377335.1	Infantis	strain	Vietnam	2017
107	GCF_013377355.1	Infantis	strain	Vietnam	2017
108	GCF_014334195.1	Enteritidis	strain	Russia	2020
109	GCF_014334255.1	SLR1		Russia	2020
110	GCF_015074885.1	Enteritidis	strain	Taiwan	2017
111	GCF_015240635.1	Enteritidis	strain	United Kingdom	1998
112	GCF_015241115.1	Enteritidis	strain	Malawi	1998
113	GCF_016127835.1	FDAARGOS		USA	2020
