# Supplementary figures and images for: Detection and Preliminary Genomic Characterization of Poultry-Derived Salmonella enterica from Southern Kazakhstan
Source: Antibiotics (Basel). 2025 Nov 25;14(12):1195. doi: 10.3390/antibiotics14121195 (PMC12729890; doi:10.3390/antibiotics14121195)

Tree scale: 0.1

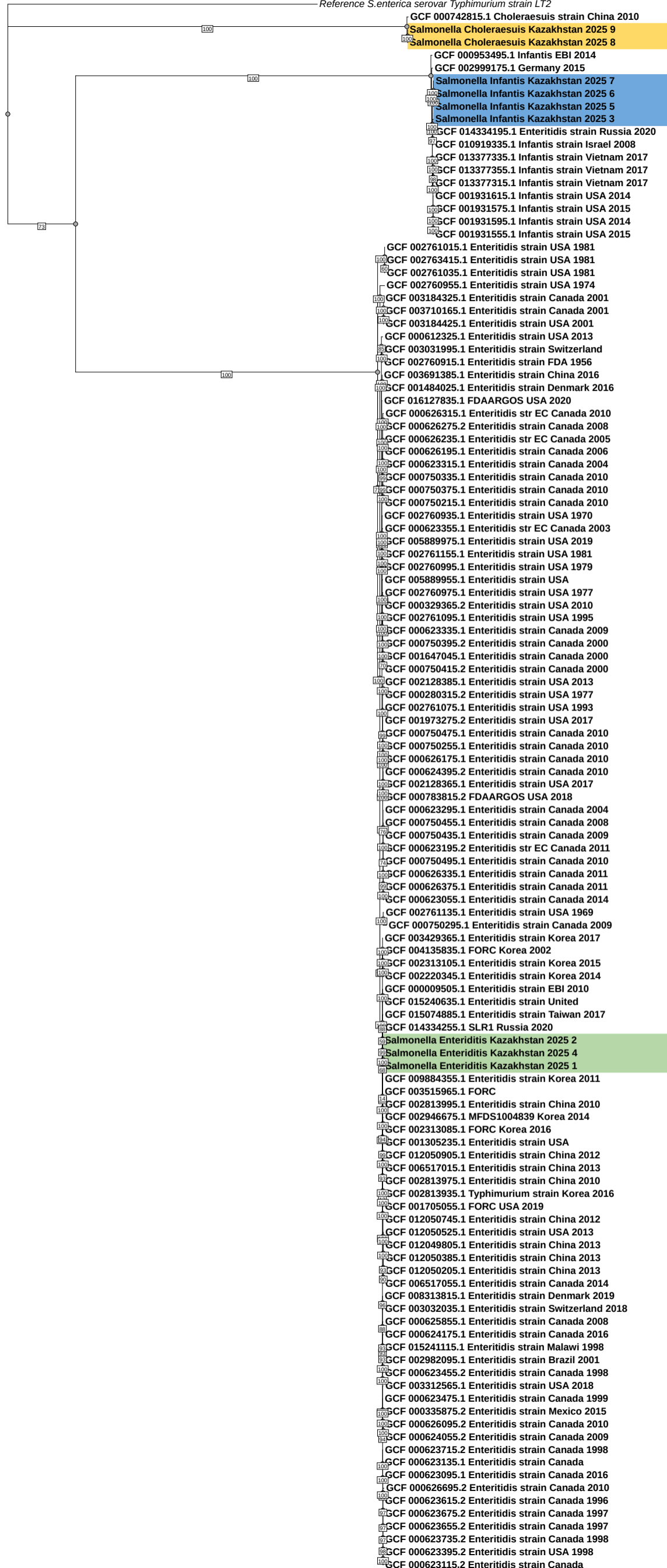

Supplement: Supplementary file 1 [file antibiotics-14-01195-s001.zip › Figure S1_TreeML_with_bs.pdf]
